# Intranasal ketamine for procedural sedation and analgesia in children: A systematic review

**DOI:** 10.1371/journal.pone.0173253

**Published:** 2017-03-20

**Authors:** Naveen Poonai, Kyle Canton, Samina Ali, Shawn Hendrikx, Amit Shah, Michael Miller, Gary Joubert, Michael Rieder, Lisa Hartling

**Affiliations:** 1 Department of Pediatrics, Schulich School of Medicine and Dentistry, London, Ontario, Canada; 2 Division of Emergency Medicine, London Health Sciences Centre, London, Ontario, Canada; 3 Children's Health Research Institute, London Health Sciences Centre, London, Ontario, Canada; 4 Department of Pediatrics, Faculty of Medicine & Dentistry, University of Alberta, Edmonton, Alberta, Canada; 5 Women and Children’s Health Research Institute, University of Alberta, Edmonton, Alberta, Canada; 6 Alberta Research Centre for Health Evidence, Department of Pediatrics, University of Alberta, Edmonton, Alberta; Nanjing University Medical School Affiliated Nanjing Drum Tower Hospital, CHINA

## Abstract

**Background:**

Ketamine is commonly used for procedural sedation and analgesia (PSA) in children. Evidence suggests it can be administered intranasally (IN). We sought to review the evidence for IN ketamine for PSA in children.

**Methods:**

We performed a systematic review of randomized trials of IN ketamine in PSA that reported any sedation-related outcome in children 0 to 19 years. Trials were identified through electronic searches of MEDLINE (1946–2016), EMBASE (1947–2016), Google Scholar (2016), CINAHL (1981–2016), The Cochrane Library (2016), Web of Science (2016), Scopus (2016), clinical trial registries, and conference proceedings (2000–2016) without language restrictions. The methodological qualities of studies and the overall quality of evidence were evaluated using the *Cochrane Collaboration’s Risk of Bias* tool, and the *Grading of Recommendations Assessment*, *Development*, *and Evaluation (GRADE)* system, respectively.

**Results:**

The review included 7 studies (n = 264) of children ranging from 0 to 14 years. Heterogeneity in study design precluded meta-analysis. Most studies were associated with a *low* or *unclear* risk of bias and outcome-specific ratings for quality of evidence were *low* or *very low*. In four of seven studies, IN ketamine provided superior sedation to comparators and resulted in adequate sedation for 148/175 (85%) of participants. Vomiting was the most common adverse effect; reported by 9/91 (10%) of participants.

**Conclusions:**

IN ketamine administration is well tolerated and without serious adverse effects. Although most participants were deemed adequately sedated with IN ketamine, effectiveness of sedation with respect to superiority over comparators was inconsistent, precluding a recommendation for PSA in children.

## Introduction

A decade of dedicated “Pain Control and Research” [1] has failed to improve the pain management of children [2] and a 2011 survey found that less than one third receive analgesia for a painful procedure [3]. Optimal pain management for children is advocated by the World Health Organization [4] and the American Academy of Pediatrics [5]. Peripheral intravenous (IV) insertion is one of the most common sources of pain in hospitalized children [6] and is consistently associated with distress [7–10]. Procedural sedation and analgesia (PSA) for maneuvers such as fracture reduction and laceration repair are common indications for IV insertion in children. The demand for PSA in children outside the operating room is increasing at a rate of 10% annually [11]. Fifteen to 27% of ED physicians report performing a fracture reduction every shift or every other shift, respectively [12], with ketamine being the most commonly used agent for PSA in children [12]. Therefore, a pain-free alternative to IV insertion is an important goal for clinicians providing sedation to children.

As a possible alternative to IVs, intranasal (IN) drugs have become increasingly popular because of ease of administration, minimal distress [3], a reduced risk of needle-stick injuries, and fewer staffing and vascular access skill requirements [13]. Demonstrating the effectiveness of IN ketamine for PSA may have widespread applicability in patients with needle-phobia, difficult IV access, in resource-limited settings, or when experience placing an IV is limited. IN ketamine has gained recent popularity for laceration repair [14] and analgesia [15] and has demonstrated good hemodynamic stability [16]. To date, no large trial or review exists upon which to base broader adoption of PSA. If we are unable to generate a meaningful summary measure of sedation supporting the use of IN ketamine, this review will highlight important features to inform future clinical trials.

Our objectives were to summarize the evidence evaluating IN ketamine versus any comparator for children who require PSA with respect to effectiveness of sedation and analgesia, ease of administration, and adverse effects. Our work has highlighted important strategies for the conduct of future clinical trials of this non-invasive approach to PSA in children.

## Material and methods

This review was conducted and reported in accordance with the Preferred Reporting Items for Systematic Review and Meta-Analysis (PRISMA) guidelines [17] (See S1 Text). The review is registered on PROSPERO (registry number CRD 420150299750) (See S2 Text).

### Search strategy

A medical librarian (SH) developed the following search strategy (see S3 Text): Ovid MEDLINE (January 1946 to August 2016); Ovid EMBASE (January 1980 to August 2016); Web of Science (August 2016); Scopus (2016); CINAHL (January 1981 to August 2016); Google Scholar (2016); Cochrane Library (August 2016). For unpublished trials, we searched clinical trial registries, research registries, and industry research databases. Key journals and conference proceedings were hand-searched from 2000 to 2016. We contacted authors for further information and checked reference lists of all included trials. The original search was completed in December 2015 and repeated in August 2016. There were no language restrictions.

### Study selection

We included all published and unpublished randomized and quasi-randomized trials comparing IN ketamine (alone or in combination) to any agent for children 0 to 19 years undergoing PSA that reported any sedation-related outcome including at least one of: duration, onset, depth, adequacy of sedation to facilitate the procedure, or adverse effects. Studies of both adult and pediatric participants were included if the authors were able to provide pediatric-specific data. We excluded sub-studies and secondary analyses of previously reported trials, studies of ketamine for psychiatric disorders, studies of IN ketamine for anesthetic premedication, and sub-dissociative dose ketamine. Two authors (NP and KC) independently screened titles and abstracts using a standardized tool. We obtained full-text copies of all studies that were not unanimously excluded and reviewed them to identify those suitable for inclusion. We resolved disagreements or uncertainty by discussion and if necessary, through arbitration with a third author (GJ).

### Data extraction and methodological quality

Two review authors (NP and KC) independently extracted the following data using a study-specific data extraction form. Data collected included age, dose, comparators, ease of administration, analgesia, adverse effects, additional sedation, proportion with adequate sedation, depth, onset, and duration of sedation. We resolved disagreements or uncertainty by consensus and if necessary, through arbitration with a third author (GJ). The primary author entered the final data into Review Manager version 5.2.3. Two reviewers (NP and KC) independently evaluated the methodological rigor of eligible studies using the *Cochrane Collaboration’s Risk of Bias* tool [18]. We followed the *Grading of Recommendations Assessment*, *Development*, *and Evaluation (GRADE)* system [19] to evaluate the strength of evidence (SOE) for patient-centered outcomes across included studies. The overall SOE was graded by two independent reviewers (NP and LH) with disagreements or uncertainty resolved through discussion and if necessary, through arbitration with a third author (GJ).

### Summary measures and synthesis of results

The primary outcome was the effectiveness of sedation. Due to differences in scales used to measure sedation, we reported the proportion of participants who’s level of sedation was adequate to facilate the procedure based on the authors’ judgment. If this information wasn’t available, we reported sedation scores. Secondary outcomes included onset and duration of sedation, ease of administration, analgesia, additional sedative medication, and adverse effects. *A priori* we considered meta-analyses if there was homogeneity in study design, dosing regimen, and indication for sedation. However, due to clinical and methodological heterogeneity across studies, we conducted a descriptive analysis of each study’s design, population, and primary outcome. When inferential statistics on the primary outcome were not performed, we analysed raw ordinal data using the Mann-Whitney U statistic (two groups) or the Kruskall-Wallis test (two or more groups). Based on a modification of the classification system of Tricco et al. [20], we categorized the results of individual studies based on the primary outcome as: *unfavorable* (effect in favor of the non-experimental comparator with p value ≤ 0.05); *neutral* (non-statistically significant difference between interventions with p value > 0.05)); *favorable* (effect in favor of the experimental agent with p value ≤ 0.05); *indeterminate* (unable to judge due to conflicting and multiple primary outcomes). We modified this classification system to categorize both non-significant positives and negatives as *neutral* [20].

## Results

**Fig 1:**
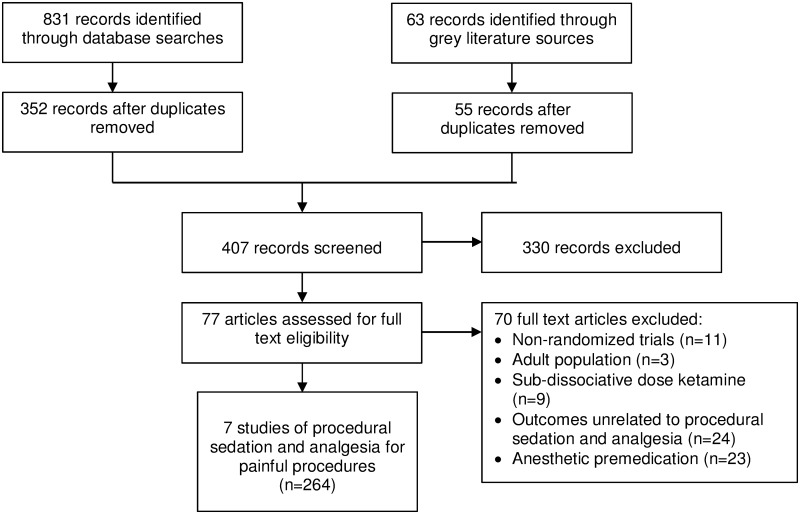
Study flow diagram.

Seven studies (264 participants) were included (Fig 1 and S2 Text) [14, 21–26]. Excessive heterogeneity in outcome measures, study design, comparators, dosing, and indications for sedation precluded meta-analysis for any of the studies.

### Included studies

The characteristics of included studies can be found in Table 1 and included children undergoing dental procedures (n = 5) [21, 22, 24–26], laceration repair (n = 1) [14], and gastric aspiration (n = 1) [23]. No quasi-randomized trials were eligible. All studies were published in English as full-text articles. We excluded one study that involved analgesia for children with fractures that reported sedation-related outcomes because it did not involve a procedure [27]. There was heterogeneity in the frequency and dose of IN ketamine. Single doses ranged from 2 to 10 mg/kg. All studies involved IN ketamine as monotherapy except two that studied IN ketamine combined with midazolam [23, 24]. The number of arms and comparison interventions were also varied and included benzodiazepines and alternative routes of ketamine delivery. The age of participants ranged from 0 to 14 years.

**Table 1:** Characteristics and results of included studies for Procedural Sedation and Analgesia (PSA).

Source, trial design, country (context)	Age range and mean age (sample size)	Comparison	Measure of effectiveness of sedation	Results	Summary
Abrams 1993 Parallel group RCT United States (dental procedures)	Range: 17–62 months; Mean: NR (n = 30)	IN Ketamine (3 mg/kg); IN Sufentanil (1 mcg/kg and 1.5 mcg/kg); IN Midazolam (0.4 mg/kg)	Depth of sedation (10-item scale)	Mean sedation score: IN ketamine 4 (range 3–6), midazolam 4 (range 2–5), high dose sufentanil 7 (range 2–9), low dose sufentanil 4 (range 3–5) (p = 0.18). Proportions adequately sedated not reported.	Neutral
Bahetwar 2011 Crossover RCT India (dental procedures)	Range: 2–6 years Mean: 4.6 years (n = 45)	IN Ketamine (6 mg/kg); IN Midazolam (0.3 mg/kg); IN Midazolam; (0.2 mg/kg) + IN Ketamine (4 mg/kg)	Proportion of participants with an “adequate” sedation on 1–5 scale	“Adequate” sedation: IN ketamine 42/45 (93%), IN midazolam 38/45 (84%), and IN midazolam + IN ketamine 40/45 (89%) (p<0.01)	Favorable for IN ketamine versus IN midazolam Neutral for IN ketamine versus IN midazolam + ketamine
Buonsenso 2014 Parallel group RCT Italy (gastric aspirates)	Range: 0–14 years Mean: 41.5 months (n = 36)	IN Ketamine (2 mg/kg) + IN Midazolam (0.5 mg/kg); Placebo (NS)	Depth of sedation using Modified Objective Pain Score and proportion requiring restraint	Mean pain score: IN ketamine + IN midazolam 3.5; placebo 7.2 (p<0.01). Level of sedation enabled gastric aspirates without physical restraint: IN ketamine + IN midazolam (18/19, 94%) vs. placebo (0/17, 0%).	Favorable for IN ketamine + midazolam (pain score and depth of sedation)
Ghajari 2015 Crossover RCT Iran (dental procedures)	Range: 3–6 years Mean: NR (n = 23)	IN Ketamine (10 mg/kg) + IN Midazolam (0.5 mg/kg); PO Ketamine (10 mg/kg) + PO Midazolam (0.5 mg/kg)	Sedation measured with Houpt scale of behavioral control and proportion with procedural success	Behavioral control significantly greater in IN group during procedure and lidocaine injection (p<0.05). Procedural success significantly greater in IN vs. oral group (97% vs. 39%) and (61% vs. 35%) at 15 and 30 minutes, respectively (p<0.05). Number of participants in each group not reported.	Favorable for IN versus PO ketamine + midazolam (procedural success)
Pandey 2011 Crossover RCT India (dental procedures)	Range: 2–6 years Mean: 4.4 years (n = 34)	IN Ketamine by nasal atomizer (6 mg/kg); IN Ketamine by nasal drops (6 mg/kg)	Proportion with “adequate” depth of sedation (5-item scale) and “successful” sedation (5-item scale)	Adequate depth of sedation with atomized ketamine (33/34, 97%) versus drops (31/34, 91%) (ns). Successful sedation with atomized ketamine (32/34, 94%) versus drops (29/34, 85%) (ns).	Neutral
Surendar 2014 Parallel RCT India (dental procedures)	Range: 4–14 years Mean: 7.3 years (n = 84)	IN Ketamine (5 mg/kg); IN Midazolam (0.2 mg/kg); IN Dexmedetomidine (1 mcg/kg); IN Dexmedetomidine (1.5 mcg/kg)	Proportion with overall “satisfactory” sedation (5-item scale) and “successful” procedure (5-item scale)	“Successful” procedure: IN ketamine (14/21, 67%), IN midazolam (13/21, 62%), IN dexmedetomidine 1 mcg/kg (17/21, 81%), IN dexmedetomidine 1.5 mcg/kg (18/21, 86%) (ns). “Satisfactory” sedation: IN ketamine (16/21, 76%), IN midazolam (15/21, 71%), I dexmedetomidine 1 mcg/kg (19/21, 91%), IN dexmedetomidine 1.5 mcg/kg (20/21, 95%) (ns).	Neutral
Tsze 2012 Parallel RCT; United States (laceration repair)	Range: 1–7 years Mean: NR (n = 12)	IN Ketamine (9 mg/kg); IN Ketamine (6 mg/kg); IN Ketamine (3 mg/kg)	Proportion with “adequate” depth of sedation using the Ramsay Sedation Score (RSS) and the Observational Scale of Behavioral Distress-Revised	3/3 (100%) patients achieved “adequate” sedation; all at a dose of 9 mg/kg. Study stopped by data safety monitoring committee because there were 9 sedation failures at doses of 3 mg/kg and 6 mg/kg.	Favorable for IN ketamine dose of 9 mg/kg vs all other doses (adequacy of sedation)

IM intramuscular; IN intranasal; IV intravenous; NR not reported; NS not significant; Observational Scale of Behavioral Distress-Revised; PO per os; PSA procedural sedation and analgesia; RCT randomized controlled trial; RSS Ramsay Sedation Score; SD standard deviation; VAS visual analog scale

### Risk of bias within studies

**Fig 2:**
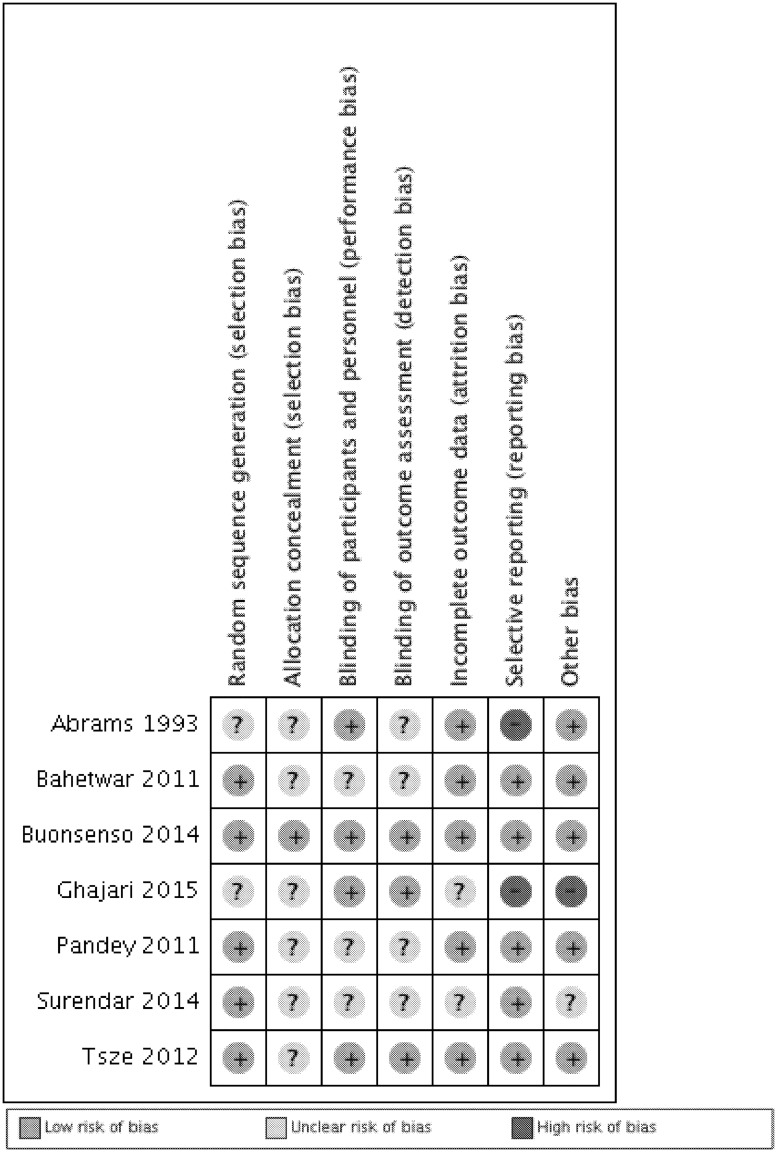
Risk of bias summary based on judgements about each item for each included study. Low risk of bias, Unclear risk of bias, High risk of bias.

Most studies were judged to have *low* or *unclear* risk of bias. Two studies were judged to have *high* risk of bias for failing to report pre-specified outcomes [21, 24] (Fig 2).

### Risk of bias across studies

Outcome-specific ratings using the GRADE system were *low* or *very low* (Table 2). SOE assessments for risk of bias were downgraded primarily due to insufficient information on randomization, allocation concealment (selection bias), or blinding (performance and detection bias). SOE assessments for consistency were downgraded for all outcomes except analgesia. SOE assessments for directness were downgraded for all outcomes except analgesia due to the use of non-validated instruments. SOE assessments for precision were downgraded for all outcomes except adverse effects due to small sample sizes (n < 200).

**Table 2:** Strength of Evidence (SOE) assessments based on *(Grading of Recommendations Assessment*, *Development*, *and Evaluation) GRADE* system.

Outcome	Number of trials Number of participants Instrument(s) Citation number	Quality assessment^a^					Importance	Strength of evidence^b^
		Risk of bias	Consistency (of effects between studies)	Directness (generalizability to population of interest)	Precision	Other considerations		
**Depth of sedation or proportion adequately sedated**								
IN ketamine vs IN midazolam vs IN ketamine + IN midazolam combination (Bahetwar 2011)	1; 45; Non-validated 4 item scales	Serious^c^	Unknown^d^	Some uncertainty about directness^e^	Imprecise	None	Critical^f^	Low
IN ketamine + IN midazolam combination vs IN saline (Buonsenso 2014)	1; 36; MOPS	None	Unknown^d^	Direct	Imprecise	None	Critical^f^	Low^g^
IN ketamine + IN midazolam combination vs PO ketamine + PO midazolam combination (Ghajari 2015)	1; 23; Non-validated behavioral scale	Serious^h^	Unknown^d^	Some uncertainty about directness^e^	Imprecise	High probability of reporting bias^i^	Critical^f^	Very low
IN ketamine vs IN ketamine (varying dose or routes) (Tsze 2012; Pandey 2011)	2; 46; Non-validated 5-item scale; RSS; OSBD-R	Serious^c^	Inconsistent	Some uncertainty about directness^e^	Precise	None	Critical^f^	Low
IN ketamine vs IN opioid (sufentanil) vs IN midazolam (Abrams 1993)	1; 30; Non-validated 10-item scale	Serious^c^	Unknown^d^	Some uncertainty about directness^e^	Precise	High probability of reporting bias^i^	Critical^f^	Low
IN ketamine vs IN dexmedetomidine vs IN midazolam (Surendar 2014)	1; 84; Non-validated 5-item scale	Serious^c^	Unknown^d^	Some uncertainty about directness^e^	Precise	None	Critical^f^	Low^g^
**Onset of sedation**								
IN ketamine alone vs comparators (Bahetwar 2011; Surendar 2014; Pandey 2011; Tsze 2012)	4; 175; RSS; OSBD-R Non-validated 3 to 5-item scales	Serious^c^	Inconsistent^n^	Some uncertainty about directness^k^	Precise	None	Less important	Low
IN ketamine + IN midazolam combination vs comparators (Buonsenso 2014)	1; 36; MOPS	Serious^c^	Unknown^d^	Some uncertainty about directness^k^	Imprecise	None	Less important	Low
**Duration of sedation**								
IN ketamine alone vs comparators (Abrams 1993; Bahetwar 2011; Pandey 2011; Surendar 2014; Tsze 2012)	5; 205; RSS; OSBD-R Non-validated 4, 5, and 10-item scales	Serious^c^^,^^j^	Inconsistent^l^	Some uncertainty about directness^k^	Precise	None	Important^m^	Low
IN ketamine + IN midazolam combination vs comparators (Buonsenso 2014; Ghajari 2015)	2; 59; MOPS; Non-validated behavioral scale	Serious^c^	Inconsistent^l^	Some uncertainty about directness^k^	Imprecise	None	Important^m^	Very low
**Analgesia**								
IN ketamine or IN ketamine + IN combination midazolam vs comparators (Buonsenso 2014; Surendar 2014)	2; 120; FLACC; MOPS	Serious^c^	Consistent	Direct	Imprecise	None	Less important	Low
**Ease of administration**								
Mucosal atomizer device (Pandey 2011)	1; 34; Non-validated 5-item scale	Serious^c^	Unknown^d^	Some uncertainty about directness^k^	Imprecise	None	Important^n^	Low
Not specified (Bahetwar 2011; Ghajari 2015)	2; 68; Not specified	Serious^c^	Inconsistent	Some uncertainty about directness^k^	Precise	None	Important^n^	Low
**Adverse effects**								
IN ketamine alone vs comparators (Abrams 1993; Bahetwar 2011; Pandey 2011; Tsze 2012)	4; 121; Frequencies	Serious^c^	Inconsistent	Some uncertainty about directness^p^	Precise	None	Critical^o^	Low
IN ketamine + IN midazolam combination vs comparators (Buonsenso 2014; Ghajari 2015)	2; 59; Incidence	Serious^c^	Inconsistent	Some uncertainty about directness^p^	Precise	None	Critical^o^	Low

FLACC Faces Legs Arms Cry Consolability; IN intranasal; MOPS Modified Objective Pain Score; OSBD-R Observational Scale of Behavioral Distress-Revised; RSS Ramsay Sedation Score; VAS Visual Analog Scale;

^a^Decrease score for: *Risk of Bias -*serious (-1) or very serious (-2) limitation to study quality; *Consistency*—important inconsistency of effects between studies (-1); *Directness*—some (-1) or major (-2) concerns about generalizability to population of interest; *Precision*–imprecise or sparse data based on number of outcome events or sample size < 200 (-1); *Other Considerations–*high probability of reporting bias (-1)

^b^SOE assessments were made using the GRADE approach as follows: Start at *high*, downgrade to *medium* due to risk of bias, downgrade to *low* due to imprecision, downgrade to *very low* due to other considerations such as risk of reporting bias. SOE categorizations are *high* quality: further research is very unlikely to change our confidence in the estimate of effect; *moderate* quality: further research is likely to have an important impact on our confidence in the estimate of effect and may change the estimate; *low* quality: Further research is very likely to have an important impact on our confidence in the estimate of effect and is likely to change the estimate; *very low* quality: Any estimate of effect is very uncertain

^c^Most or all studies report insufficient details of at ≥ 1 of: randomization, allocation concealment (selection bias), or blinding (performance and detection bias)

^d^Unable to assess given only one study

^e^Use of a non-standardized tool to determine level and adequacy of sedation in at least one study limits the degree to which the results can be applied broadly

^f^This outcome was deemed *critical* because suboptimal sedation leads to pain and distress and can lengthen the duration of procedural sedation, increasing the risk of morbidity

^g^Downgraded because only a single study contributed to the comparisons

^h^Method of randomization and allocation concealment not reported (selection bias); participant numbers not reported; not all outcomes reported (reporting bias)

^i^Authors did not report all pre-specified outcomes (reporting bias)

^j^At least one study in which complete follow-up of participants was not specified (attrition bias)

^k^Use of a non-standardized tools to measure this outcome limits the degree to which the results can be applied broadly

^l^Downgraded for consistency due to the large range in this outcome, which was in turn likely due to heterogeneity in measurement instruments and dose

^m^This outcome was deemed *important* (rather than *critical*) because it is a determinant of the expected duration of monitoring. While it may not affect morbidity and mortality, it may impact staffing resources, making it important to health care providers

^n^This outcome was deemed *important* (rather than *critical*) because although it may not affect morbidity and mortality, the willingness of a patient to accept IN therapy may impact staffing resources and the willingness of the clinician to use it

^o^This outcome was deemed *critical* because although adverse effects were generally minor and transient, they may affect morbidity and mortality

^p^Validated tools not consistently used to ascertain or quantify emergence agitation

### Onset, duration, and depth of sedation

Depth of sedation was reported in all studies. There was heterogeneity in the time interval from administration of the intervention to recording this outcome. Two studies [14, 23] used validated measures of sedation; either the Ramsay Sedation Score (RSS) [28], the Observational Scale of Behavioral Distress-Revised [29], or the Modified Objective Pain Score [30]. In both of these studies, the outcome assessor was blinded [14, 23].

Six of seven studies of PSA reported the adequacy of sedation and overall, 148/175 participants (85%) were deemed adequately sedated with IN ketamine to facilitate the procedure [14, 21–23, 25, 26]. Four studies were deemed “favorable” because they reported adequate sedation in significantly more participants than the comparator [14, 22–24]. They involved IN ketamine as monotherapy (6 and 9 mg/kg) [14, 22] or co-administered with IN midazolam (0.5 mg/kg) [23, 24]. Among the two studies that used validated measures of sedation, Tsze et al. found that only at a dose of 9 mg/kg did IN ketamine produce “adequate” sedation in all three participants undergoing laceration repair [14]. Buonsenso et al. found IN ketamine 2 mg/kg in combination with IN midazolam provided adequate sedation for gastric aspirates (Table 1).

Five studies reported onset of sedation [14, 22, 23, 25, 26], with means ranging from 3.6 to 11.6 minutes. Duration of sedation was reported in all studies and when IN ketamine was used as monotherapy, the means ranged from 7 to 69 minutes.

### Co-administration of IN ketamine with IN midazolam

IN ketamine was co-administered with IN midazolam in two studies [23, 24]. Both provided “favorable” sedation.

### Requirement of additional sedative medication

Provision of additional (rescue) medication for sedation was not reported in any study.

### Analgesia

Analgesia was reported in two studies. Surendar et al. included children undergoing dental extraction [26] and used a validated measure of pain (Faces Legs Activity Cry Consolability scale [31]). Buonsenso et al. studied children undergoing gastric aspirates [23] also used a validated measure (Modified Objective Pain Score [32]). Both studies found IN ketamine to provide adequate analgesia.

### Ease of IN administration

Three studies reported administration with a mucosal atomizer device (MAD) [14, 23, 25]. The remainder described only a “syringe” or provided no description. Ease of IN ketamine administration was reported in three studies [22, 24, 25] but the proportion of participants in which administration was well tolerated (71/79, 90%) was reported in only two studies [22, 25]. Pandey et al. reported IN ketamine was significantly better tolerated than ketamine drops using a standardized tool for behavioral response in 34 participants [25].

### Adverse effects

Adverse effects were reported in six studies [14, 21–25]. This was most commonly nausea and vomiting and reported in four studies [14, 22, 24, 25]. Only Buonsenso et al. reported emergence agitation [23] and did not use a validated scale to measure it’s degree. Overall, vomiting and emergence agitation was reported in 9/91 (10%) and 6/57 (11%) participants who received IN ketamine, respectively, either alone or in combination with midazolam. Abrams et al. was the only study to report transient, spontaneously-resolving oxygen desaturations in two of ten participants [21].

## Discussion

This systematic review assessed the effectiveness of IN ketamine for sedation across all randomized trials of children undergoing PSA. IN ketamine administration was generally well tolerated with minor adverse effects. Although most participants were deemed adequately sedated, superiority of IN ketamine over comparators with respect to effective sedation was inconsistent. Most studies had a *low* or *unclear* risk of bias and the quality of evidence was *low* or *very low*. At present, there is insufficient evidence to recommend IN ketamine for PSA in children in clinical settings.

One of our primary objectives was to review the evidence for IN ketamine in PSA. Although most participants achieved adequate sedation, studies were inconsistent for sedation effectiveness versus comparators. This may have been due to heterogeneity in dosing, non-experimental comparators, scales, and indications. Only Tzse et al. used a validated tool and a blinded outcome assessor to measure sedation [14]. Despite the study’s small sample size, their findings suggest that 9 mg/kg is required for effective sedation for laceration repair. Importantly, there were no trials exploring IN ketamine for fracture reduction, the most common pediatric indication for PSA [33, 34]. There were also no trials comparing IN ketamine to IV sedatives, making it difficult to ascertain whether the level of sedation produced by IN ketamine can obviate the need for an IV. As such, no recommendations can be made for IN ketamine in PSA in children.

The need for additional (rescue) sedative medication was not described in any reviewed study. For clinicians, this outcome is important because rescue sedation, most likely administered through an IV, would offset the benefits of IN administration. Therefore, the need for rescue sedation should be included as an outcome in future trials of sedation effectiveness.

Wide ranges were found in reporting onset and duration of sedation, likely owing to varied instruments and definitions of this interval. Onset of sedation in studies reporting this outcome was appreciably longer (11 minutes) than what has been described for IV ketamine (1 minute) [35]. This is consistent with IN ketamine’s time to peak plasma concentration of 18 [36] to 21 minutes [37]. Duration of sedation is an outcome important to patients, administrators and clinicians. Yet, only one study used a validated measure of sedation and a blinded outcome assessor. The authors reported a range of 42 to 69 minutes with a dose of 9 mg/kg [14]. This data was obtained from only three participants and future investigations of IN ketamine must report this clinically relevant outcome.

The co-administration of benzodiazepines with ketamine has been traditionally recommended as a strategy to mitigate emergence reactions [38]. In contrast to adults [39], a large meta-analysis has not supported a role for midazolam in reducing emergence reactions in children [40]. Consistent with this, only one study in our review reported emergence agitation in children receiving IN ketamine co-administered with IN midazolam. The authors reported a greater incidence of emergency agitation (6/57, 11%) [23] than previously described (1.5%) [40]. Despite their study’s methodological limitations, Bahetwar et al. compared IN ketamine co-administered with IN midazolam to each intervention alone [22] and their findings suggested that the addition of IN midazolam to IN ketamine confers no benefit to depth of sedation. Co-administration of a benzodiazepine has been identified as a risk factor for airway complications in children [41] and adults [42]. As such, the co-administration of IN ketamine and IN midazolam cannot be recommended for PSA in children.

Our review found that despite higher per kilogram doses of IN ketamine, the incidence of the most frequent adverse effect, vomiting, was consistent with a previous report [40]. This was not unexpected given that there is no evidence of a dose relationship with IV ketamine [40]. Importantly however, the frequency of emergence agitation in our review [23] was more than three-fold greater than previously reported for IV ketamine [40]. The data from our review must be interpreted with caution however, as Buonsenso et al. reported cases of emergence agitation in children undergoing gastric aspirates [23] using a non-standardized definition of this outcome. Future trial designs should utilize validated measures to describe emergence agitation and its clinical significance. The lack of serious adverse events in our review is consistent with Green et al.’s meta-analysis of IV ketamine in children where the authors found serious, albeit transient complications (laryngospasm and apnea), in 0.3% and 0.8%, respectively [41]. The number of participants in our review was insufficient to detect these serious outcomes. Patient safety is quite likely the most important variable for health care providers considering the use of IN ketamine over the IV route. Given the relative infrequency of these potentially serious complications, long-term surveillance studies may be the best approach to accurately estimate this risk.

Only three studies reported that interventions were in fact atomized [14, 23, 25]. Fluid volumes in excess of 0.3 mL that are instilled into the nasopharynx without an MAD may result in excess drug deposition into the pharynx [43]. Although a non-atomized approach has been described [3], it may result in unpredictable drug deposition, raising the possibility that sedative effects may be due to oral rather than transmucosal absorption.

Analgesia was not the primary focus of our review. However, this parameter is a salient feature of an ideal monotherapy for PSA [44]. Buonsenso et al. [23] reported that IN ketamine was associated with a reduction in pain scores that exceeded the minimal clinically important difference on the Modified Objective Pain Score [30]. Surendar et al. reported similar findings but the clinical significance of the analgesic effect was uncertain and change scores were not reported [26]. Although limited, evidence from this review pertaining to analgesia was consistent and is in line with what is known about ketamine’s analgesic properties [45]. Consistency in analgesic efficacy across studies using validated instruments suggests that IN ketamine can be recommended as an effective analgesic for the indications reviewed. A notable caveat is that our search did not include all studies reporting analgesia. As a result, specific recommendations for indications and dosing regimen are beyond the scope of this review.

### Limitations

The primary limitation of our review was our decision to summarize results based on the authors’ judgement of the adequacy of sedation. This measure was inherently subjective, given the lack of a consistent, objective definition of adequacy, even among studies using validated instruments. The Canadian Anesthesiologists’ Society has recommended the use of instruments with identifiable endpoints such as the RSS [44], used in several reviewed studies. However, a blinded outcome assessor, remote from the clinical encounter, and a tool that does not involve physically stimulating the participant, such as the Dartmouth Operative Conditions Scale [46], would provide a more non-intrusive and therefore objective way to measure sedation.

## Conclusions

In this systematic review of seven studies, IN ketamine produced sedation adequate enough to perform the procedure under study. However, the superiority of IN ketamine over comparators with respect to effective sedation was inconsistent. IN ketamine was well tolerated in most participants without serious adverse effects. Reviewed studies were limited by poor methodological rigor, small sample sizes, inconsistent results, and limited generalizability with respect to effectiveness of sedation. These factors preclude a recommendation for the use of IN ketamine for PSA in children in clinical settings. No study explored the utility of IN ketamine for common procedures such as fracture reduction or compared IN ketamine to IV sedatives. The adoption of IN over IV ketamine by clinicians is therefore contingent on the findings of larger, high quality trials that employ validated instruments and are adequately powered to detect clinically meaningful differences in outcomes such as analgesia, depth and duration of sedation.

## Supporting information

S1 Text (PDF)

S2 Text (PDF)

S3 Text (PDF)

S4 Text (PDF)
