# Supplementary material for: Intranasal ketamine for procedural sedation and analgesia in children: A systematic review
Source: PLoS One. 2017 Mar 20;12(3):e0173253. doi: 10.1371/journal.pone.0173253 (PMC5358746; doi:10.1371/journal.pone.0173253)
Supplement: S2 Text — (PDF) [file pone.0173253.s002.pdf]

PROSPERO International prospective register of systematic reviews

Review title and timescale

1 Review title
Give the working title of the review. This must be in English. Ideally it should state succinctly the interventions or exposures being reviewed and the associated health or social problem being addressed in the review.
Intranasal ketamine for procedural sedation and analgesia in children: a systematic review

2 Original language title
For reviews in languages other than English, this field should be used to enter the title in the language of the review. This will be displayed together with the English language title.

3 Anticipated or actual start date
Give the date when the systematic review commenced, or is expected to commence.
03/12/2015

4 Anticipated completion date
Give the date by which the review is expected to be completed.
01/07/2016

5 Stage of review at time of this submission
Indicate the stage of progress of the review by ticking the relevant boxes. Reviews that have progressed beyond the point of completing data extraction at the time of initial registration are not eligible for inclusion in PROSPERO. This field should be updated when any amendments are made to a published record.

The review has not yet started ×

Review stage	Started	Completed
Preliminary searches	Yes	Yes
Piloting of the study selection process	Yes	Yes
Formal screening of search results against eligibility criteria	Yes	Yes
Data extraction	Yes	Yes
Risk of bias (quality) assessment	Yes	Yes
Data analysis	Yes	Yes

Provide any other relevant information about the stage of the review here.

Review team details

6 Named contact
The named contact acts as the guarantor for the accuracy of the information presented in the register record.
Dr Poonai

7 Named contact email
Enter the electronic mail address of the named contact.
poonai@hotmail.com

8 Named contact address
Enter the full postal address for the named contact.
84 Harris Road Delaware, Ontario, Canada N0L 1E0

9 Named contact phone number
Enter the telephone number for the named contact, including international dialing code.
5196945309

10 Organisational affiliation of the review
Full title of the organisational affiliations for this review, and website address if available. This field may be completed as 'None' if the review is not affiliated to any organisation.
Western University

Website address:
None

11 Review team members and their organisational affiliations

Give the title, first name and last name of all members of the team working directly on the review. Give the organisational affiliations of each member of the review team.

Title	First name	Last name	Affiliation
Dr	Naveen	Poonai	Western University
Mr	Kyle	Canton	Western University
Dr	Samina	Ali	University of Edmonton
Dr	Sylvie	LeMay	University of Montreal
Dr	Michael	Rieder	Western University

12 Funding sources/sponsors

Give details of the individuals, organizations, groups or other legal entities who take responsibility for initiating, managing, sponsoring and/or financing the review. Any unique identification numbers assigned to the review by the individuals or bodies listed should be included.

None

13 Conflicts of interest

List any conditions that could lead to actual or perceived undue influence on judgements concerning the main topic investigated in the review.

Are there any actual or potential conflicts of interest?

None known

14 Collaborators

Give the name, affiliation and role of any individuals or organisations who are working on the review but who are not listed as review team members.

Title	First name	Last name	Organisation details

Review methods

15 Review question(s)

State the question(s) to be addressed / review objectives. Please complete a separate box for each question.

Is intranasal ketamine efficacious for sedation in children undergoing painful procedures?

Is intranasal ketamine an efficacious analgesic in children undergoing painful procedures?

Is intranasal ketamine associated with greater adverse drug events in children undergoing painful procedures?

Is intranasal ketamine associated with greater time to recovery in children undergoing painful procedures?

Is intranasal ketamine associated with a greater requirement for adjunctive sedation or analgesia in children undergoing painful procedures?

Is intranasal ketamine associated with greater patient or caregiver satisfaction in children undergoing painful procedures?

16 Searches

Give details of the sources to be searched, and any restrictions (e.g. language or publication period). The full search strategy is not required, but may be supplied as a link or attachment.

EMBASE 1947-2015 MEDLINE 1946-2015 Web of Science Google Scholar Grey literature: - Abstracts from medical conferences (2011-2015) - Clinical trial registries - Bibliographies No language restrictions

17 URL to search strategy

If you have one, give the link to your search strategy here. Alternatively you can e-mail this to PROSPERO and we will store and link to it.

I give permission for this file to be made publicly available
Yes

18 Condition or domain being studied
Give a short description of the disease, condition or healthcare domain being studied. This could include health and wellbeing outcomes.
Painful conditions requiring procedural sedation and analgesia with intranasal ketamine.

19 Participants/population
Give summary criteria for the participants or populations being studied by the review. The preferred format includes details of both inclusion and exclusion criteria.
Inclusion criteria: Children aged 0-19 years who have a painful condition for which they underwent procedural sedation and analgesia with intranasal ketamine. Exclusion criteria: IN ketamine for non-painful procedures (eg. MRI imaging) or IN ketamine for non-sedating therapy (eg. pre-anesthetic induction or exclusively pain management) No exclusion criteria.

20 Intervention(s), exposure(s)
Give full and clear descriptions of the nature of the interventions or the exposures to be reviewed
Intranasal ketamine administered to children aged 0-19 years with painful conditions for the purpose of procedural sedation and analgesia. Any dose, frequency, duration, setting, and comparator.

21 Comparator(s)/control
Where relevant, give details of the alternatives against which the main subject/topic of the review will be compared (e.g. another intervention or a non-exposed control group).
Any other agent for procedural sedation and analgesia.

22 Types of study to be included
Give details of the study designs to be included in the review. If there are no restrictions on the types of study design eligible for inclusion, this should be stated.
All randomized and quasi-randomized trials published in any language that have studied intranasal ketamine in children who require procedural sedation and analgesia for a painful condition.

23 Context
Give summary details of the setting and other relevant characteristics which help define the inclusion or exclusion criteria.
Not applicable.

24 Primary outcome(s)
Give the most important outcomes.
Degree of sedation and analgesia.

Give information on timing and effect measures, as appropriate.
Outcomes must be related to the intervention being explored.

25 Secondary outcomes
List any additional outcomes that will be addressed. If there are no secondary outcomes enter None.
Frequency of adverse events, caregiver satisfaction, frequency of requirement for adjunctive sedation therapy, and duration of sedation.

Give information on timing and effect measures, as appropriate.
Outcomes must be related to the intervention being explored.

26 Data extraction (selection and coding)
Give the procedure for selecting studies for the review and extracting data, including the number of researchers involved and how discrepancies will be resolved. List the data to be extracted.
Two independent reviewers will select studies for review using a librarian's search results. Data extraction will be performed by both reviewers independently using a standardized data abstraction form. Discrepancies will be resolved by consensus and arbitration by a third co-investigator if necessary. Data to be extracted include: - study design - publication status - participant age and sex - primary and secondary outcomes (above) - reporting parameters - risk of bias

27 Risk of bias (quality) assessment

State whether and how risk of bias will be assessed, how the quality of individual studies will be assessed, and whether and how this will influence the planned synthesis.

Risk of bias will be assessed by both reviewers independently using the Cochrane Collaboration Risk of Bias tool.

28 Strategy for data synthesis

Give the planned general approach to be used, for example whether the data to be used will be aggregate or at the level of individual participants, and whether a quantitative or narrative (descriptive) synthesis is planned. Where appropriate a brief outline of analytic approach should be given.

Data will be synthesized using aggregate data for studies that included exclusively paediatric patients. For those studies that included a mixed population, the authors will be contacted to provide patient-level data. Meta-analyses will be performed if there is minimal heterogeneity with respect to how the primary outcomes are reported. In the case where no meta-analyses are performed, a narrative (descriptive) synthesis will be performed.

29 Analysis of subgroups or subsets

Give any planned exploration of subgroups or subsets within the review. ‘None planned’ is a valid response if no subgroup analyses are planned.

Subgroup analysis will be performed based on the dose of intranasal ketamine being used.

Review general information

30 Type and method of review

Select the type of review and the review method from the drop down list.

Intervention, Systematic review

31 Language

Select the language(s) in which the review is being written and will be made available, from the drop down list. Use the control key to select more than one language.

English

Will a summary/abstract be made available in English?

Yes

32 Country

Select the country in which the review is being carried out from the drop down list. For multi-national collaborations select all the countries involved. Use the control key to select more than one country.

Canada

33 Other registration details

Give the name of any organisation where the systematic review title or protocol is registered together with any unique identification number assigned. If extracted data will be stored and made available through a repository such as the Systematic Review Data Repository (SRDR), details and a link should be included here.

34 Reference and/or URL for published protocol

Give the citation for the published protocol, if there is one.

Give the link to the published protocol, if there is one. This may be to an external site or to a protocol deposited with CRD in pdf format.

I give permission for this file to be made publicly available

Yes

35 Dissemination plans

Give brief details of plans for communicating essential messages from the review to the appropriate audiences.

The results of the review will be presented at a national conference and published in a peer-reviewed journal

Do you intend to publish the review on completion?

Yes

36 Keywords

Give words or phrases that best describe the review. (One word per box, create a new box for each term)
Intranasal ketamine

Paediatric

Pain

Procedural sedation

37 Details of any existing review of the same topic by the same authors

Give details of earlier versions of the systematic review if an update of an existing review is being registered, including full bibliographic reference if possible.

38 Current review status

Review status should be updated when the review is completed and when it is published.
Completed but not published

01/10/2016

39 Any additional information

Provide any further information the review team consider relevant to the registration of the review.

40 Details of final report/publication(s)

This field should be left empty until details of the completed review are available.
Give the full citation for the final report or publication of the systematic review.
Give the URL where available.
