# Supplementary material for: Intranasal ketamine for procedural sedation and analgesia in children: A systematic review
Source: PLoS One. 2017 Mar 20;12(3):e0173253. doi: 10.1371/journal.pone.0173253 (PMC5358746; doi:10.1371/journal.pone.0173253)
Supplement: S3 Text — (PDF) [file pone.0173253.s003.pdf]

Appendix

Clinical Trial Registries

International Standard Randomised Controlled Trials Number (ISRCTN) Register
United Kingdom Clinical Research Network
Clinicaltrials.gov
Australian New Zealand Clinical Trials Registry
European Union Clinical Trials Register
World Health Organization International Clinical Trials Registry Platform

Research Registries
National Research Registry Archive
Health Services Research Projects in Progress (HSRproj)
National Institutes of Health Reporter

Industry Results Databases
PhRMA Clinical Study Results Database
International Federation of Pharmaceutical Manufacturers and Associations (IFPMA) Clinical Trials Portal
Eli Lilly™ and Company Clinical Trial Registry
Roche™ Clinical Study Register
GlaxoSmithKline™ Clinical Study Register

Conference Proceedings
European Society of Regional Anaesthesia 34th Annual Congress
Australian Society of Anaesthetists and New Zealand Society of Anaesthesia Combined Scientific Meeting
New England Society of Anaesthesiologists Annual Meeting And Fall Conference
European Society for Paediatric Anaesthesiology Annual Congress in association with Turkish Association Of Anaesthetics And Resuscitation
Association Of Anaesthetists Of Great Britain And Ireland Annual Congress
Anaesthetic Trauma and Critical Care
American Society for Pediatric Anesthesia Annual Meeting
American Society of Anesthesiologists Annual Meeting
American Society of Regional Anesthesia and Pain Medicine Annual Pain Medicine Meeting
Canadian Anesthesiologists' Society Annual Meeting
The Congress of the European Pain Federation
World Federation of Societies of Intensive and Critical Care Medicine Congress
European Society of Intensive Care Medicine 28th Annual Congress 2015
Anaesthetic Trauma and Critical Care
Critical Care Canada Forum
Canadian Association of Emergency Physicians
Society of Academic Emergency Medicine
American College of Emergency Physicians

Australasian College for Emergency Medicine
National Capital Conference on Emergency Medicine
International Federation on Emergency Medicine
European Society of Emergency Medicine
International Conference on Emergency Medicine
American Academy of Pediatrics
Canadian Paediatric Society
Pediatric Academic Societies

<u>Ovid Search Strategy</u>
Database(s): Ovid MEDLINE(R) In-Process & Other Non-Indexed Citations, Ovid MEDLINE(R) Daily and Ovid MEDLINE(R) 1946 to Present
Search Strategy:

#	Searches	Results
1	Administration, Intranasal/	11734
2	intranasal.mp.	22134
3	intra-nasal.mp.	263
4	(nasal adj3 (administration$ or drug$ or instillation$ or medication$)).mp.	2045
5	1 or 2 or 3 or 4	23199
6	Ketamine/	9827
7	"2 (2 chlorophenyl) 2 (methylamino)cyclohexanone".mp.	3
8	"2 (2 chlorophenyl) 2 methylaminocyclohexanone".mp.	0
9	"2 (methylamino) 2 (2 chlorophenyl)cyclohexanone".mp.	0
10	"2 (ortho chlorophenyl) 2 (methylamino)cyclohexanone".mp.	0
11	"2 (ortho chlorophenyl) 2 methylaminocyclohexanone".mp.	0
12	"2 (ortho chlorophenyl) 2 methylaminocyclohexanone hydrochloride".mp.	0
13	"2 methylamino 2 (2 chlorophenyl)cyclohexanone".mp.	0
14	"2 ortho chlorophenyl 2 methylaminocyclohexanone".mp.	0
15	anesject.mp.	0
16	calipsol.mp.	18
17	calypsol.mp.	37
18	ci 581.mp.	147
19	ci581.mp.	3
20	cl 369.mp.	2
21	cl369.mp.	0
22	cn 52,372 2.mp.	0
23	cn 52372 2.mp.	0
24	cn 523722.mp.	0
25	cn52,372 2.mp.	0
26	cn52372 2.mp.	0

27	cn523722.mp.	0
28	imalgene.mp.	1
29	kalipsol.mp.	27
30	katamine.mp.	4
31	keta-hameln.mp.	0
32	ketaject.mp.	2
33	ketalar.mp.	205
34	ketalin.mp.	0
35	ketamax.mp.	0
36	ketamine hcl.mp.	112
37	ketamine hydrochloride.mp.	561
38	ketaminol vet.mp.	1
39	ketanest.mp.	33
40	ketased.mp.	2
41	ketaset.mp.	14
42	ketaved.mp.	0
43	ketavet.mp.	1
44	ketmin.mp.	0
45	ketoject.mp.	0
46	ketolar.mp.	13
47	narkamon.mp.	3
48	narketan.mp.	0
49	soon-soon.mp.	0
50	tekam.mp.	1
51	velonarcon.mp.	1
52	vetalar.mp.	8
53	ketamine.mp.	15111
54	or/6-53	15297
55	5 and 54	115

Database(s): Ovid EMBASE 1980 to 2016 Week 32
Search Strategy:

#	Searches	Results
1	intranasal.mp.	26069
2	intra-nasal.mp.	402
3	(nasal adj3 (administration$ or drug$ or instillation$ or medication$)).mp.	2812
4	Ketamine/	26689
5	"2 (2 chlorophenyl) 2 (methylamino)cyclohexanone".mp.	6
6	"2 (2 chlorophenyl) 2 methylaminocyclohexanone".mp.	2

7	"2 (methylamino) 2 (2 chlorophenyl)cyclohexanone".mp.	0
8	"2 (ortho chlorophenyl) 2 (methylamino)cyclohexanone".mp.	0
9	"2 (ortho chlorophenyl) 2 methylaminocyclohexanone".mp.	0
10	"2 (ortho chlorophenyl) 2 methylaminocyclohexanone hydrochloride".mp.	0
11	"2 methylamino 2 (2 chlorophenyl)cyclohexanone".mp.	0
12	"2 ortho chlorophenyl 2 methylaminocyclohexanone".mp.	0
13	anesject.mp.	0
14	calipsol.mp.	33
15	calypsol.mp.	52
16	ci 581.mp.	174
17	ci581.mp.	2
18	cl 369.mp.	4
19	cl369.mp.	0
20	cn 52,372 2.mp.	0
21	cn 52372 2.mp.	0
22	cn 523722.mp.	0
23	cn52,372 2.mp.	0
24	cn52372 2.mp.	0
25	cn523722.mp.	0
26	imalgene.mp.	43
27	kalipsol.mp.	21
28	katamine.mp.	6
29	keta-hameln.mp.	0
30	ketaject.mp.	38
31	ketalar.mp.	933
32	ketalin.mp.	3
33	ketamax.mp.	1
34	ketamine hcl.mp.	132
35	ketamine hydrochloride.mp.	587
36	ketaminol vet.mp.	5
37	ketanest.mp.	304
38	ketased.mp.	4
39	ketaset.mp.	227
40	ketaved.mp.	7
41	ketavet.mp.	41
42	ketmin.mp.	3
43	ketoject.mp.	2
44	ketolar.mp.	42

45	narkamon.mp.	17
46	narketan.mp.	23
47	soon-soon.mp.	0
48	tekam.mp.	5
49	velonarcon.mp.	8
50	vetalar.mp.	130
51	ketamine.mp.	29411
52	or/4-51	29642
53	intranasal drug administration/	11799
54	1 or 2 or 3 or 53	27940
55	52 and 54	247

CINAHL Search Strategy

Tuesday, July 19, 2016 10:46:07 AM

#	Query	Results
S7	S3 AND S6	28
S6	S4 OR S5	1,953
S5	(MH "Administration, Intranasal")	1,359
S4	intranasal OR intra-nasal OR "nasal administration" OR "nasal drug administration" OR "nasal instillation" OR "nasal medication"	1,953
S3	S1 OR S2	1,452
S2	ketamine OR anesject OR calipsol OR calypsol OR ci 581 OR ci581 OR cl 369 OR cl369 OR cn 52,372 2 OR cn 52372 2 OR cn 523722 OR cn52,372 2 OR cn52372 2 OR cn523722 OR imalgene OR kalipsol OR katamine OR keta-hameln OR ketaject OR ketalar OR ketalin OR ketamax OR ketamine hcl OR ketamine hydrochloride OR ketaminol vet OR ketanest OR ketased OR ketaset OR ketaved OR ketavet OR ketmin OR ketoject OR ketolar OR narkamon OR narketan OR soon-soon OR tekam OR velonarcon OR vetalar	1,452
S1	(MH "Ketamine")	1,193

Web of Science Search Strategy

TS=(ketamine OR anesject OR calipsol OR calypsol OR ci 581 OR ci581 OR cl 369 OR cl369 OR cn 52,372 2 OR cn 52372 2 OR cn 523722 OR cn52,372 2 OR cn52372 2 OR cn523722 OR imalgene OR kalipsol OR katamine OR keta-hameln OR ketaject OR ketalar OR ketalin OR ketamax OR ketamine hcl OR ketamine hydrochloride OR ketaminol vet OR ketanest OR ketased OR ketaset OR ketaved OR ketavet OR ketmin OR ketoject OR ketolar OR narkamon OR narketan OR soon-soon OR tekam OR velonarcon OR vetalar) AND TS=(intranasal OR intra-nasal OR "nasal administration" OR "nasal drug administration" OR "nasal instillation" OR "nasal medication")

Scopus Search Strategy

TITLE-ABS-KEY(ketamine) AND TITLE-ABS-KEY(intranasal OR intra-nasal OR "nasal administration" OR "nasal drug administration" OR "nasal instillation" OR "nasal medication")

EMBASE Search Strategy

Database(s): EMBASE Classic+EMBASE 1947 to 2015 July 30
Search Strategy:

#	Searches	Results
1	intranasal.mp.	27863
2	intra-nasal.mp.	450
3	(nasal adj3 (administration$ or drug$ or instillation$ or medication$)).mp.	2999
4	Ketamine/	29118
5	"2 (2 chlorophenyl) 2 (methylamino)cyclohexanone".mp.	6
6	"2 (2 chlorophenyl) 2 methylaminocyclohexanone".mp.	2
7	"2 (methylamino) 2 (2 chlorophenyl)cyclohexanone".mp.	0
8	"2 (ortho chlorophenyl) 2 (methylamino)cyclohexanone".mp.	0
9	"2 (ortho chlorophenyl) 2 methylaminocyclohexanone".mp.	0
10	"2 (ortho chlorophenyl) 2 methylaminocyclohexanone hydrochloride".mp.	0
11	"2 methylamino 2 (2 chlorophenyl)cyclohexanone".mp.	0
12	"2 ortho chlorophenyl 2 methylaminocyclohexanone".mp.	0
13	anesject.mp.	0
14	calipsol.mp.	33
15	calypsol.mp.	52
16	ci 581.mp.	250
17	ci581.mp.	4
18	cl 369.mp.	4
19	cl369.mp.	0
20	cn 52,372 2.mp.	0
21	cn 52372 2.mp.	0
22	cn 523722.mp.	0
23	cn52,372 2.mp.	0
24	cn52372 2.mp.	0
25	cn523722.mp.	0
26	imalgene.mp.	50
27	kalipsol.mp.	21
28	katamine.mp.	9
29	keta-hameln.mp.	0
30	ketaject.mp.	68

31	ketalar.mp.	1309
32	ketalin.mp.	3
33	ketamax.mp.	1
34	ketamine hcl.mp.	168
35	ketamine hydrochloride.mp.	729
36	ketaminol vet.mp.	5
37	ketanest.mp.	354
38	ketased.mp.	4
39	ketaset.mp.	252
40	ketaved.mp.	7
41	ketavet.mp.	41
42	ketmin.mp.	3
43	ketoject.mp.	2
44	ketolar.mp.	54
45	narkamon.mp.	17
46	narketan.mp.	23
47	soon-soon.mp.	0
48	tekam.mp.	5
49	velonarcon.mp.	8
50	vetalar.mp.	169
51	ketamine.mp.	31940
52	or/4-51	32181
53	exp Pain/	967680
54	pain.mp.	904298
55	53 or 54	1169758
56	intranasal drug administration/	13046
57	1 or 2 or 3 or 56	29933
58	exp infant/ or (infant$ or infancy or newborn$ or baby$ or babies or neonat$ or preterm$ or prematur$ or postmatur$).mp. or exp child/ or (child$ or schoolchild$ or school child& or school age or preschool$ or kid or kids or toddler$).mp. or adolescent/ or adolescence/ or (adoles$ or teen$ or boy$ or girl$).mp. or "minor (person)"/ or exp puberty/ or (pubert$ or pubescen$ or prepubescen$).mp. or exp pediatrics/ or (paediatric$ or pediatric$ or peadiatric$ or Neonatolog$ or Perinatolog$).mp. or exp school/ or (nursery school$ or kindergar$ or primary school$ or secondary school$ or elementary school$ or high school$ or highschool$).mp.	4166000
59	52 and 55 and 57 and 58	49
