# Supplementary material for: Intranasal ketamine for procedural sedation and analgesia in children: A systematic review
Source: PLoS One. 2017 Mar 20;12(3):e0173253. doi: 10.1371/journal.pone.0173253 (PMC5358746; doi:10.1371/journal.pone.0173253)
Supplement: S4 Text — (PDF) [file pone.0173253.s004.pdf]

PRISMA 2009 Flow Diagram

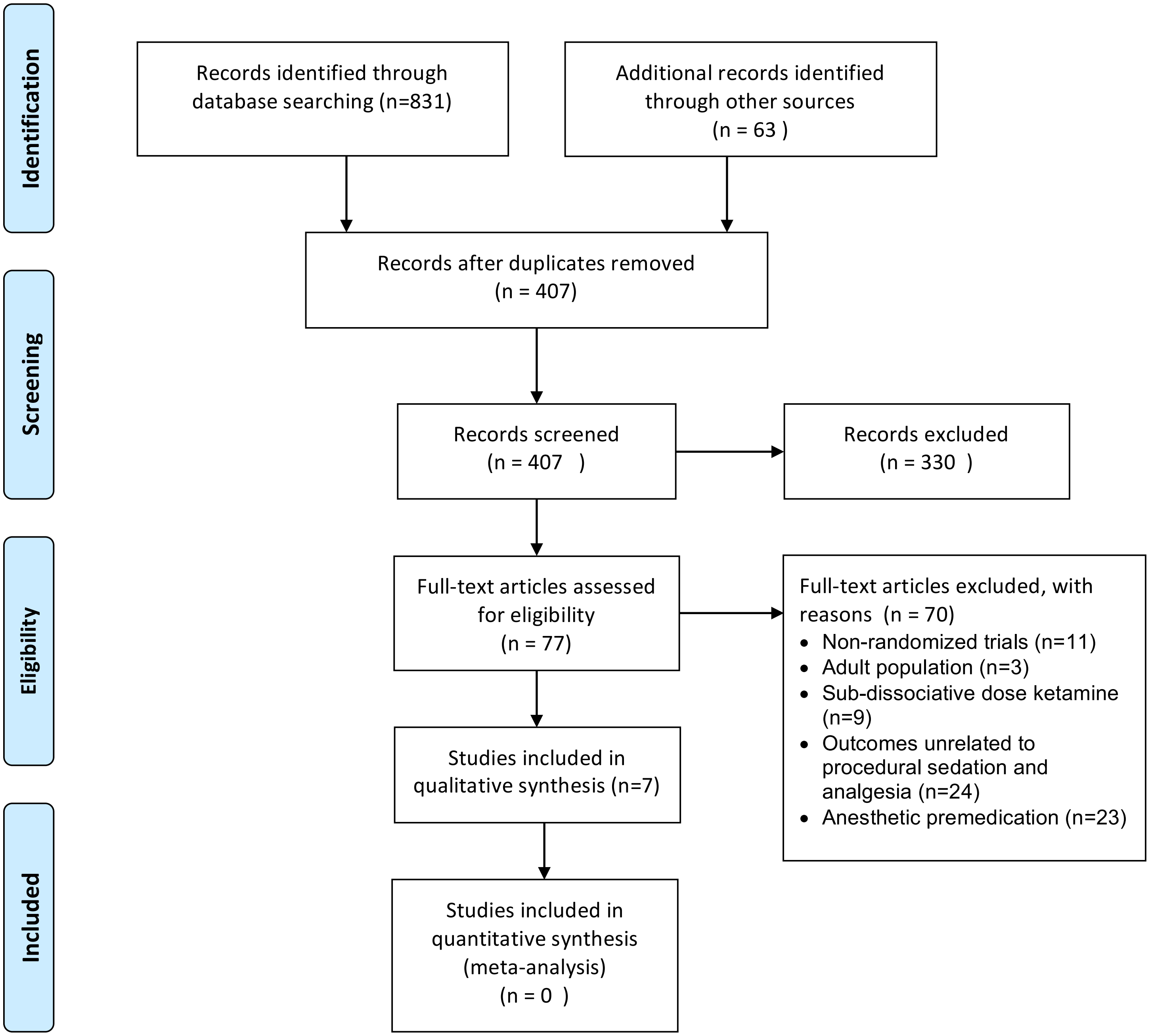

From: Moher D, Liberati A, Tetzlaff J, Altman DG, The PRISMA Group (2009). *P*referred *R*eporting *I*tems for *S*ystematic Reviews and *M*eta-*A*nalyses: The PRISMA Statement. PLoS Med 6(6): e1000097. doi:10.1371/journal.pmed1000097

For more information, visit www.prisma-statement.org.
